# MafB silencing in macrophages does not influence the initiation and growth of lung cancer induced by urethane

**DOI:** 10.17179/excli2017-325

**Published:** 2017-06-20

**Authors:** Takako Nemoto, Yoko Shibata, Sumito Inoue, Akira Igarashi, Yoshikane Tokairin, Keiko Yamauchi, Tomomi Kimura, Masamichi Sato, Kento Sato, Hiroshi Nakano, Shuichi Abe, Michiko Nishiwaki, Maki Kobayashi, Sujeong Yang, Yukihiro Minegishi, Kodai Furuyama, Hiroyoshi Machida, Isao Kubota

**Affiliations:** 1 Department of Cardiology, Pulmonology, and Nephrology, Yamagata University School of Medicine, Yamagata, Japan

**Keywords:** MafB, lung cancer, urethane, tumor-associated macrophages, gene targeted mouse

## Abstract

An increased number of tumor-associated macrophages (TAMs) that exhibit the M2 macrophage phenotype is related to poorer prognosis in cancer patients. MafB is a transcription factor regulating the differentiation of macrophages. However, involvement of MafB for the development of TAMs is unknown. This study was designed to investigate the role of MafB in a murine urethane-induced lung cancer model. Urethane was injected intraperitoneally into wild-type and dominant-negative MafB transgenic mice. Twenty-four weeks later, mice were sacrificed and their lungs removed for pathological analysis. The numbers and mean areas of lung cancer were evaluated. In addition, the numbers of Mac-3-positive macrophages were evaluated in each tumor. The numbers and mean areas of lung cancer induced by urethane administration were not significantly different between wild-type and dominant-negative MafB transgenic mice. The numbers of TAMs in lung cancer tissue were not significantly different between the two groups. MafB silencing using dominant-negative MafB did not influence the initiation and growth of lung cancer in mice exposed to urethane. These data suggest that MafB may not be related to the development of TAMs.

## Introduction

Lung cancer is the first and third leading cause of death in men and women in Japan, respectively. Advances involving many new molecularly-targeted drugs, such as epidermal growth factor receptor tyrosine kinase inhibitors and anaplastic lymphoma kinase inhibitors, improve the therapeutic outcome of lung cancer (Janne et al., 2015[12]; Mok et al., 2009[17]; Shaw et al., 2013[27]). In addition, immune checkpoint inhibitors, such as antibodies against programed cell death (PD)-1 and PD-ligand (L)1, prolong the survival of patients with lung cancer (Brahmer et al., 2015[3]; Garon et al., 2015[6]; Herbst et al., 2016[10]). Specifically, the binding of PD-1 on cytotoxic T cells, and PD-L1 on tumor cells, reduces the cytotoxic activity of T cells against tumor cells. Antibodies against PD-1 and PD-L1 prevent this immune tolerance and suppress tumor growth in cancer patients (Park et al., 2016[22]). These inhibitors are the first immunotherapies showing significant clinical effects against lung cancer (Park et al., 2016[22]). 

Macrophages are the most abundant immune cells in the alveolar space of non-diseased human lungs. Macrophages monitor the occurrence of tumors and eliminate neoplastic cells. In contrast, tumor-associated macrophages (TAMs) play important roles in tumor growth and metastasis by producing growth and angiogenic factors (Pollard, 2004[23]). Thus, an increased number of TAMs in tumors is associated with poor prognosis in cancer patients (Kim et al., 2015[14]; Shigeoka et al., 2013[28]; Yang et al., 2015[31]).

We reported previously that MafB gene-targeted mice exhibited significant changes in alveolar macrophage numbers, the nuclear compartment, cellular shape, surface marker expression, and phagocytic function (Sato-Nishiwaki et al., 2013[26]). However, the role of MafB in TAMs has not been investigated. In this study, we evaluated whether MafB silencing influenced the initiation and growth of lung cancer induced by urethane using previously established transgenic (Tg) mice that specifically express dominant-negative (DN) MafB in macrophages under control of the macrophage scavenger receptor enhancer-promoter.

## Materials and Methods

### Mice

As described previously, we established macrophage scavenger receptor enhancer-promoter DN MafB transgenic (DN-MafB Tg) mice on the C57/BL6 background in which the activity of MafB was suppressed only in macrophages (Sato-Nishiwaki et al., 2013[26]). Female wild-type (WT) mice, purchased from CLEA Japan (Tokyo, Japan), were used as controls in these experiments. Mice were housed in a facility with a 12/12-h light/dark cycle and were given free access to water and standard rodent chow. The rooms were kept free from any pathogens. Natural survival curves were not different between WT and DN-MafB mice. The study was approved by the Committee for Animal Experimentation, Yamagata University School of Medicine, and was carried out in accordance with the Declaration of Helsinki.

### Urethane-induced lung cancer

Female 10- to 12-week-old WT and DN-MafB Tg mice (mean body weight: 18.8 ± 0.9 g vs. 18.2 ± 0.8 g, *P* = 0.29) were injected intraperitoneally with 1 g/kg urethane (Sigma-Aldrich, St. Louis, MO, USA) dissolved in 0.9 % saline (Salazar-Degracia et al., 2016[25]). After 24 weeks, the mice were sacrificed for lung fixation. Mean body weight at 24-weeks were 23.0 ± 0.7 g in WT mice and 22.3 ± 0.7 g in DN-MafB Tg mice (P =0.45).

### Histopathological examination

Lungs were fixed by the intratracheal instillation of 4 % buffered formalin at a constant pressure of 25 cm H_2_O. After removal of the lungs and overnight fixation in 4 % formalin, paraffin-embedded lung blocks were prepared. These lung-sections (3 µm thickness) were stained with hematoxylin and eosin. Twenty-four weeks after urethane treatment, proliferative lung lesions were detected using a light microscope. We evaluated the number and area of the tumors in each mouse in sections sliced to maximize the left and right lung areas. The areas were evaluated by Image J software (National Institutes of Health, Bethesda, MD, USA). There was no difference in the area of the sliced lungs between WT and DN-MafB mice (data not shown).

### Immunohistochemical staining

Lung-sections were also stained with an anti-Mac-3 antibody (BD Biosciences, San Diego, CA, USA; diluted 1:100), and then were stained with an anti-rat IgG-HRP antibody (Stressgen, CA, USA; diluted 1:200).

### Statistical analysis

Data are expressed as means ± standard deviations. Differences between means were assessed using Welch's *t*-test. Survival curves were analyzed by the log-rank test. *P* values < 0.05 were considered statistically significant. All statistical analyses were performed using JMP version 11.0 software (SAS Institute, Cary, NC, USA).

## Results

### The numbers and areas of urethane-induced lung cancer

**Figure 1:**
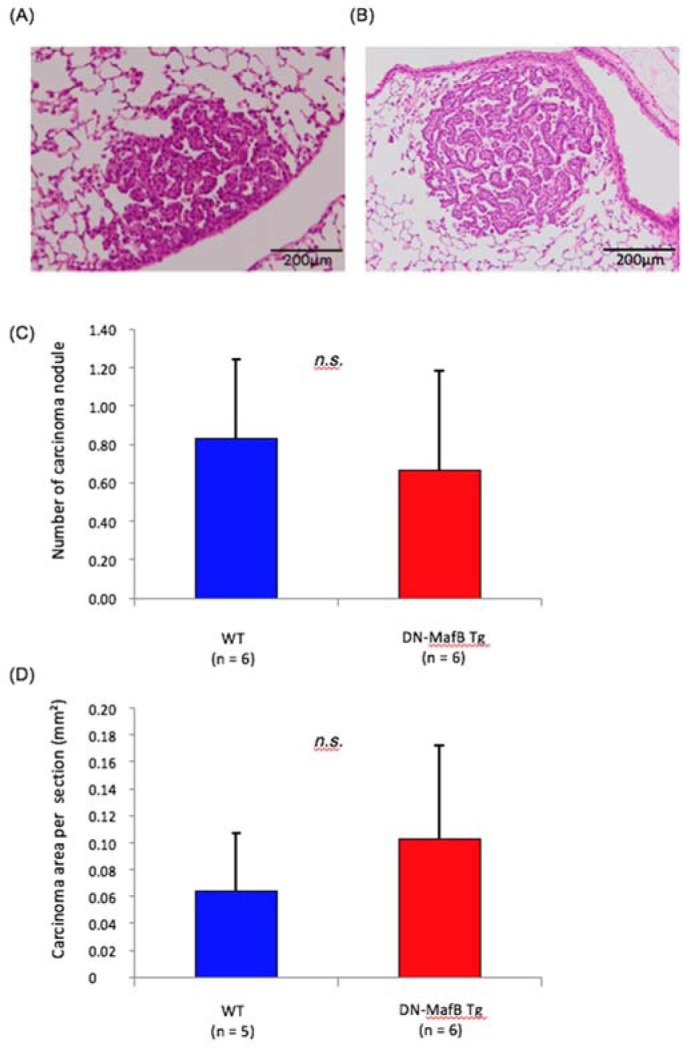
The numbers and mean areas of lung cancer after urethane treatment of wild-type (WT) and dominant-negative (DN)-MafB transgenic (Tg) mice. Representative images of adenocarcinomas in lung tissue from (A) WT and (B) DN-MafB Tg mice stained with hematoxylin and eosin. (C) The number of carcinoma nodules per section from the lungs of WT and DN-MafB Tg mice was not significantly different between the two groups. (D) The mean carcinoma area per lung section was not significantly different between the two groups. Data are expressed as means ± SD (P > 0.05).

Representative cross-sectional images of lung tissue from urethane-treated WT and DN-MafB Tg mice are shown in Figures 1A and B(Fig. 1), respectively. Some hyperplastic and adenocarcinoma lesions were detected. The numbers of carcinoma nodules per section were not significantly different between WT and DN-MafB Tg mice (Figure 1C(Fig. 1)). The numbers of hyperplasic lesions per section were also not significantly different between the two groups (WT: 1.0 ± 0.46/section, DN-MafB: 0.83 ± 0.46/section, *P* = 0.80). In addition, the mean tumor areas were not different between the two groups (Figure 1D(Fig. 1)).

### The numbers of TAMs in urethane-induced lung cancer tissue

**Figure 2:**
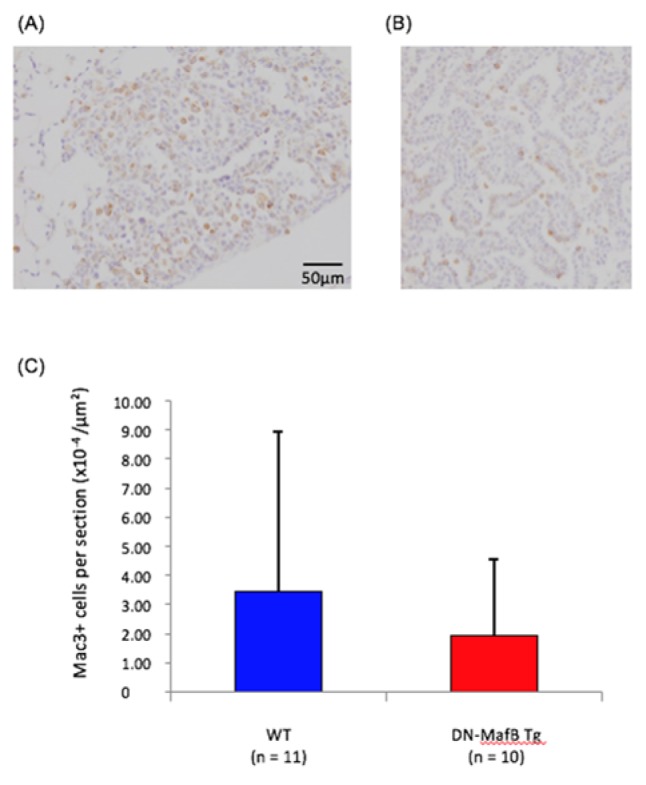
The numbers of tumor-associated macrophages in urethane-induced lung cancer of wild-type (WT) and dominant-negative (DN)-MafB transgenic (Tg) mice. Representative images of adenocarcinomas in lung sections of (A) WT and (B) DN-MafB Tg mice stained with the Mac-3 antibody. (C) The numbers of Mac-3-positive cells in urethane-induced lung cancer lesions of WT and DN-MafB Tg mice were not significantly different between the two groups. Data are expressed as means ± SD (P > 0.05).

The numbers of TAMs in adenocarcinoma lesions were compared between WT and DN-MafB Tg mice. Representative images of WT (Figure 2A(Fig. 2)) and DN-MafB Tg (Figure 2B(Fig. 2)) mice are shown. The numbers of Mac-3 positive macrophages were not significantly different between the two groups (Figure 2C(Fig. 2)).

## Discussion

In this study, we evaluated the impact of silencing MafB in macrophages on the initiation and growth of lung cancer induced by urethane. The total tumor area after urethane treatment did not differ significantly between WT and DN-MafB Tg mice. In addition, the number of TAMs in the lung cancer lesions was not significantly different between WT and DN-MafB Tg mice (Figure 1(Fig. 1)).

Typically, macrophages play an important role in innate immunity that monitors and exterminates malignant neoplasms. However, an increase in the number of TAMs in tumors is related to poor prognosis in cancer patients (Kim et al., 2015[14]; Shigeoka et al., 2013[28]; Yang et al., 2015[31]). These dichotomous effects arise because TAMs, M2-type macrophages, produce tumor growth factors such as epidermal growth factor, angiogenic factors such as vascular endothelial growth factor, and metastasis promoting factors such as matrix metalloproteinase (Qian and Pollard, 2010[24]).

In our previous studies, MafB, a regulator of macrophage differentiation, was shown to suppress apoptosis and control phagocytosis of macrophages (Machiya et al., 2007[16]; Nemoto et al., 2017[19]; Sato-Nishiwaki et al., 2013[26]). It is assumed that MafB affects macrophage-mediated tumor immunity by altering their antigen presenting ability, which may affect the initiation and promotion of lung cancer in mouse models. In addition, peritoneal macrophages recovered from DN-MafB/ ApoE knockout mice have increased expression of M1 macrophage markers after lipopolysaccharide stimulation (Hasegawa et al., 2016[8]). Therefore, in our gene-targeted mouse, it was assumed that M2 polarization in TAMs was insufficient. Furthermore, it was expected that the initiation and promotion of urethane-induced lung cancer would be suppressed. However, contrary to our expectation, MafB suppression did not affect these processes.

In recent years, immunotherapy has attracted attention as a cancer treatment. Especially, antibodies against PD-1 and PD-L1 have prominent effects in various malignant tumors (Brahmer et al., 2010[4]; Garon et al., 2015[6]; Herbst et al., 2014[11]; Topalian et al., 2012[30]). PD-1 is expressed on cytotoxic T lymphocytes and prevents the immune response of these cells to tumor cells by binding to PD-L1 expressed on tumor cells. Therefore, important roles of cytotoxic T lymphocytes in tumor immunity have been proven clinically. 

Other studies indicate that TAMs contribute to the promotion of cancer. For example, there is a connection between an increase in the number of these cells and poor prognosis in many human cancers (Ahn et al., 2010[1]; Steidl et al., 2010[29]). Therefore, targeting TAMs may be a novel strategy for treating cancer. Inhibiting the recruitment of monocytes to tumor tissue is one clinical tactic (Yang and Zhang, 2017[32]). Antibody therapies targeting factors related to the accumulation of monocytes in tissues, such as CCL2/CCR2, and adhesion to vascular endothelial cells, such as CD11b, have also been attempted in animal cancer experiments and clinical studies of human cancer (Ahn et al., 2010[1]; Nywening et al., 2016[21]).

Inhibiting the activity of TAMs may suppress the progression of cancer. Development of antitumor drugs targeting the CSF1/CSF1 receptor, mannose receptor CD206, and scavenger receptor A is proceeding (He et al., 2012[9]; Movahedi et al., 2012[18]; Neyen et al., 2013[20]). In particular, trabectedin decreases the number of TAMs in tumor tissue by a selective cytotoxic effect on monocytes and macrophages (Allavena et al., 2005[2]; Germano et al., 2013[7]). 

Macrophages have the potential to change their phenotype in the tumor microenvironment. Therefore, reprogramming TAMs to an antitumor phenotype is another therapeutic approach (Yang and Zhang, 2017[32]). Antitumor macrophages are expected to scavenge and destroy tumor cells. Pseudomonas aeruginosa mannose-sensitive hemagglutinin for malignant pleural effusion has converted CD163+ TAMs to M1 macrophages. In addition, reprogramming CD163+ TAMs is a potential therapeutic strategy for malignant pleural effusion (Yang et al., 2015[31]; Yang and Zhang, 2017[32]). 

β-Glucan is a yeast-derived polysaccharide that has been suggested to differentiate TAMs into M1 macrophages (Chan et al., 2009[5]). Immunotherapy using β-glucan is already under investigation in a phase I clinical trial in patients with neuroblastoma (Kushner et al., 2014[15]). In addition, reprogramming of TAMs to the M1 phenotype by manganese nanoparticles, CD40, and toll-like receptor agonists is also possible (Yang and Zhang, 2017[32]).

In contrast to the positive effect of suppressing TAMs in cancer treatment, the initiation and growth of urethane-induced lung tumors were not influenced by the expression of DN-MafB in macrophages. Although we confirmed suppression of MafB activity in bone marrow-derived macrophages in the gene-targeted mice used in this study, MafB suppression in TAMs was not confirmed. The fraction of TAMs in the lungs was too small to investigate the activity of MafB in this model (Figure 2(Fig. 2)). Because DN-MafB is expressed under control of the macrophage scavenger receptor promoter in this mouse, the expression level of DN-MafB might be insufficient for suppression of MafB activity in TAMs. The MafB knockout mouse dies soon after birth (Kataoka et al., 1994[13]). Thus, alternative experimental approaches are not currently available.

In conclusion, MafB silencing using DN-MafB does not influence the initiation and growth of lung cancer in mice exposed to urethane. Thus, MafB may not be related to the development of TAMs.

## Conflict of interests

The authors declare that there is no conflict of interests regarding the publication of this paper.

## Funding

This work was supported by grants from the Japan Society for the Promotion of Science.

## Acknowledgements

The authors thank Emiko Nishidate, Hiroko Sasaki and Eiji Tsuchida for their invaluable technical assistance.
